# Encoding contexts are incidentally reinstated during competitive retrieval and track the temporal dynamics of memory interference

**DOI:** 10.1093/cercor/bhab529

**Published:** 2022-02-01

**Authors:** Inês Bramão, Jiefeng Jiang, Anthony D Wagner, Mikael Johansson

**Affiliations:** Department of Psychology, Lund University, Lund SE-221 00, Sweden; Department of Psychological and Brain Sciences, University of Iowa, Iowa 52242-1407, USA; Department of Psychology, Stanford University, CA 94305, USA; Department of Psychology, Wu Tsai Neurosciences Institute, Stanford University, Stanford, CA 94305, USA; Department of Psychology, Lund University, Lund SE-221 00, Sweden

**Keywords:** electroencephalography (EEG), episodic memory, memory reactivation, multivariate-pattern analysis (MVPA)

## Abstract

The ability to remember an episode from our past is often hindered by competition from similar events. For example, if we want to remember the article a colleague recommended during the last lab meeting, we may need to resolve interference from other article recommendations from the same colleague. This study investigates if the contextual features specifying the encoding episodes are incidentally reinstated during competitive memory retrieval. Competition between memories was created through the AB/AC interference paradigm. Individual word-pairs were presented embedded in a slowly drifting real–word-like context. Multivariate pattern analysis (MVPA) of high temporal-resolution electroencephalographic (EEG) data was used to investigate context reactivation during memory retrieval. Behaviorally, we observed proactive (but not retroactive) interference; that is, performance for AC competitive retrieval was worse compared with a control DE noncompetitive retrieval, whereas AB retrieval did not suffer from competition. Neurally, proactive interference was accompanied by an early reinstatement of the competitor context and interference resolution was associated with the ensuing reinstatement of the target context. Together, these findings provide novel evidence showing that the encoding contexts of competing discrete events are incidentally reinstated during competitive retrieval and that such reinstatement tracks retrieval competition and subsequent interference resolution.

## Introduction

Episodic memory allows us to travel back in time to revisit our past (Tulving 1983). However, access to a particular past event is often hindered by retrieval competition, which occurs when retrieval cues not only overlap with the sought-after event but also with other similar events (Anderson 1974; Mensink and Raaijmakers 1988). Context reinstatement theories (e.g., Estes 1955; Polyn et al. 2009; Howard and Kahana 2012; Howard 2017; Yonelinas et al. 2019; Kahana 2020) describe how our ongoing experience is represented in the brain by the binding of slowly-drifting contextual information to discrete events. Accordingly, context retrieval during episodic remembering serves to organize our personal past. However, little is known about the role of context retrieval during competitive remembering. It has recently been hypothesized that contextual information can act as a form of cognitive control that mitigates the effects of memory interference in working memory (Beukers et al. 2021). In this study, we leveraged multivariate pattern analyses (MVPA) of high temporal-resolution electroencephalographic (EEG) data to measure the reactivation of contextual details during long-term episodic remembering. Our data provide novel evidence that contextual information is incidentally reinstated during the competitive retrieval of discrete events and that such reinstatement tracks competition and interference resolution.

Episodic memory is by definition context dependent (Estes 1955; Godden and Baddeley 1975; Polyn et al. 2009; Howard 2017) and previous studies indicate that episodic remembering involves the reinstatement of the items and the contextual features of the original event. Accordingly, it has been shown that retrieval success covaries with the neural reinstatement of goal-relevant memory traces (Polyn et al. 2005; Staresina et al. 2012; Gordon et al. 2014; Jafarpour et al. 2014), that retrieval cues trigger reinstatement of contextual features that guide behavior (Jiang, Bramão, et al. 2020a; Jiang, Wang, et al. 2020b), and that retrieval may also reactivate goal-irrelevant contextual features (Manning et al. 2011; Diana et al. 2013; Gershman et al. 2013; Miller et al. 2013; Staudigl et al. 2015; Folkerts et al. 2018; Herweg, Solomon, et al. 2020b). Recently, Herweg, Sharan, et al. (2020a), Herweg, Solomon, et al. (2020b) have shown that spatial context reinstatement may precede item retrieval and that theta oscillatory activity, in the medial temporal lobe, coordinates the episodic retrieval of item and context. However, these previous studies have investigated contextual reinstatement in noncompetitive retrieval paradigms.

Thus far, little is known about incidental context reinstatement during competitive retrieval. The reinstatement of contextual information during competitive retrieval may play an important role by separating competing discrete events and thereby reducing memory interference. Interestingly, Manning et al. (2016) found less context reinstatement after a directed-forgetting instruction, suggesting that intentional forgetting actively washes out the contextual features of the past event (Sahakyan and Kelley 2002). In this study, we elucidate how the reinstatement of contextual information occurs during competitive retrieval of discrete events. Previous work has shown that memory interference is associated with ambiguous neural reinstatement patterns and with the engagement of frontoparietal control processes that resolve competition between target memories (Wimber et al. 2009; Kuhl et al. 2011; Wimber et al. 2015). EEG data indicate that memory interference is associated with positive-going frontal slow waves (Johansson et al. 2007; Hellerstedt and Johansson 2014) and with increased frontal theta activity (Hanslmayr et al. 2010; Staudigl et al. 2010; Waldhauser et al. 2012), which are likely triggered by the simultaneous reactivation of target and competing memory traces. In this study, we use MVPA of EEG data recorded during memory retrieval to investigate the concurrent and incidental reactivation of the contexts in which target and competing discrete events were embedded during encoding.

**Figure 1:**
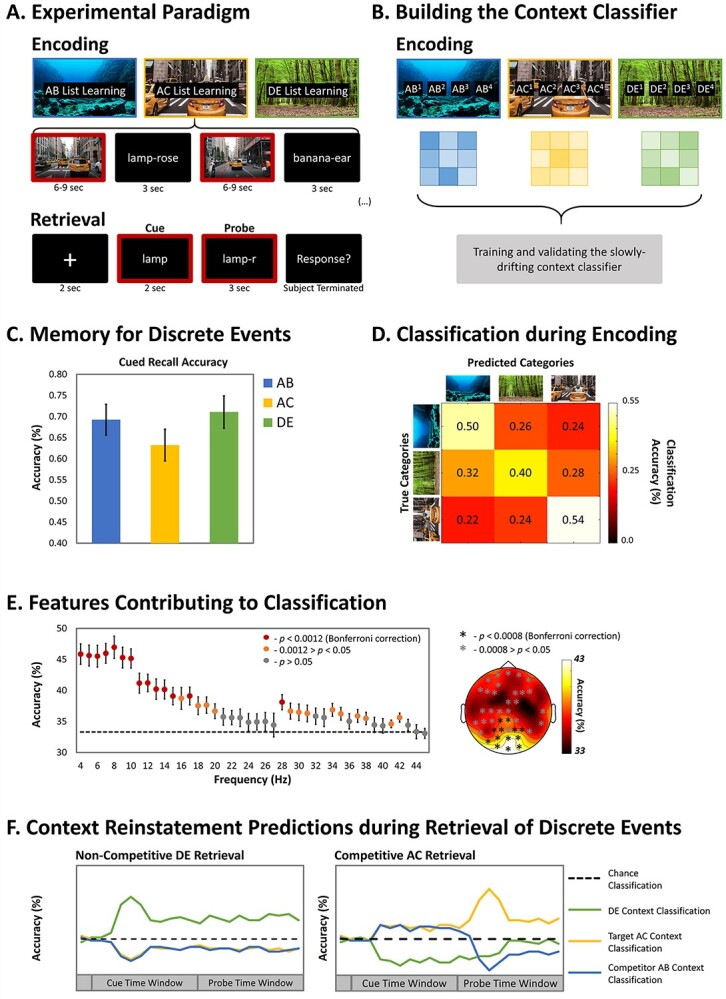
(*A*) Competition between memories was created through an AB/AC associative memory paradigm. At retrieval, participants were first presented with the cue word. Then, the first letter of the target associate was presented, and participants were asked to retrieve that associate (the E, or either the B or the C). (*B*) We trained classifiers to discriminate patterns of brain activity associated with the context movie at encoding (outlined in red in *A*). (*C*) Behavioral results. Error bars represent standard error (SE) of the mean. (*D*) The confusion matrix shows classification performance at encoding. (*E*) The contribution of the frequency band (left) and channel (right) to encoding context classification. (*F*) The pattern classifier, trained at encoding, was applied at retrieval to track context reinstatement (in the time widows outlined in red in *A*). Plotted is the predicted classification accuracy during retrieval for noncompetitive DE retrieval and competitive AC retrieval.

Memory interference was experimentally induced in an adapted AB/AC paradigm (e.g., Kuhl et al. 2011). Participants first encoded novel cue–associate word-pairs (AB) and later overlapping associations formed by pairing repeated cues with novel associates (AC). Nonoverlapping novel word-pairs (DE) served as a noncompetitive control condition. To simulate the rich and complex nature of real-world episodes, the discrete word-pairs were embedded in one of three multimodal movie contexts (i.e., first-person movie of being in an underwater, a forest, or a city environment). Several discrete word-pairs were presented within the same extended movie context, allowing the simulation of the slowly drifting nature of real-word contexts (Stark et al. 2018). Critically, the AB, AC, and DE word-pairs were encoded with different movies, enabling the quantification of context reinstatement at retrieval (Fig. 1*A*). Participants were instructed to intentionally learn the paired associates whereas the context was incidental to the memory task (Smith and Vela 2001; Stark et al. 2018). A pattern classifier was trained during encoding to discriminate the oscillatory brain activity patterns associated with the different dynamic contexts (Fig. 1*B*). At retrieval, participants were presented with a word-cue (either the A or the D of a specific pair), followed by the presentation of a first-letter probe, indicating which of the associates (either the B or C, or the E) to selectively recall. The classifier was applied over the time course of retrieval to quantify and reveal the temporal dynamics of target and competitor context reinstatement (Fig. 1*A*,*F*).

We first examined if the original dynamic encoding contexts (movies), despite being incidental to the memory task, are reinstated during the retrieval of the discrete events (word-pairs). If so, we predicted that noncompetitive retrieval, in contrast to the competitive retrieval, would be associated with the reinstatement of the target context already in the word-cue time window. The word-cue, in noncompetitive retrieval, is only encountered in a single encoding event; thus it can promptly trigger retrieval of the sought-after memory trace. After having established a way of quantifying the incidental reinstatement of the encoding context, we turn to our primary aim and measure the temporal dynamics of target and competitor context reinstatement during competitive retrieval. We predicted that 1) retrieval competition would be associated with the simultaneous reactivation of target and competing contexts or with no reactivation because both contexts compete for reactivation and neither wins and 2) that subsequent interference resolution would be associated with the emergence of a stronger target relative to competitor context reinstatement in the probe time window (Fig. 1*A*,*F*).

## Materials and Methods

### Participants

Thirty human participants took part in the study (average 22 years old, range 19–29; 17 female). All participates were right handed, native Swedish speakers, and reported no history of neurologic or psychiatric diseases. Participants received a movie ticket as a compensation for volunteering to participate in the study. The study was conducted in accordance with the Swedish Act concerning the Ethical Review of Research involving Humans. Participants gave written informed consent, and the study followed the local ethical guidelines at Lund University.

### Stimuli Materials

One-hundred and forty-four word-triplets were used in the experiment. The words were all concrete words selected from a Swedish language corpus (Borin et al. 2012). The words in a triplet contained different initial letters and did not have any obvious association with each other. The triplets were divided into two lists, matched by length and frequency (all *P*s > 0.4), of 72 triplets each. Each list was assigned to a specific experimental condition (competitive AB and AC, and noncompetitive DE). Three versions of each list were created in which the status of each word in the triplet was rotated across cue, target, and competitor. To ensure that differences between conditions are not due to differences in the word material, the assignment of the word lists was counterbalanced across experimental conditions and participants.

Three first-person perspective movie contexts were used in the experiment: 1) diving underwater, 2) walking in a forest, and 3) driving in New York City. To optimize multivariate pattern classification, we used contexts that were perceptually and semantically different from each other.

### Experimental Design and Procedures

The complete experiment comprised 18 blocks, each including an encoding phase, a 30-s counting-backward distractor task, and a retrieval phase. In the encoding phase (Fig. 1*A*), participants were presented with word-pair associates embedded in a movie. Each word-pair was presented for 3 s in between movie segments that lasted for 6–9 s. To create strong memory representations, participants were asked to intentionally learn the paired associates and visualize them in the context movies. Notice that the movie context was incidental to the memory task (i.e., the association between context and word-pairs was not an explicit learning goal) (Smith and Vela 2001; Stark et al. 2018).

First participants were presented with four novel cue-associates (AB word-pairs). To create memory interference, participants subsequently encoded word-pairs that contain a repeated cue paired with a novel associate (AC word-pairs). Finally, a separate set of four novel cue-associates (noncompetitive DE word-pairs), not followed by overlapping pairs, was also presented. Critically, to be able to track the reactivation of target versus incidental features, the movies associated with AB, AC, and DE word-pairs were different. The assignment of movie to experimental conditions (AB, AC, and DE) was counterbalanced within participant across the different experimental blocks. Thus, each movie was used equally often in the three experimental conditions over the whole experiment. This ensures that the MVPA-based classification is based on reactivation of the movie contexts rather than on the demands of the AB/AC/DE experimental conditions. The presentation order of the AB, AC, and DE word-pairs was counterbalanced across blocks, with the constraint that AB word-pairs always preceded AC word-pairs.

In the retrieval phase, memory for all 12 presented word-pairs was tested in a cued-recall task (i.e., the four ABs, four ACs, and four DEs). Participants were first tested on all the four DE word-pairs, together with two AB and another two AC randomly selected word-pairs. The retrieval order of these first eight word-pairs was random. Importantly, the presented EEG analysis was constrained to the retrieval of these first eight word-pairs to ensure that each cue was used only once. At the end of the retrieval block, memory performance for the four competitor associations was also tested (i.e., the AB/AC target memory test was followed by an AC/AB competitor memory test). The retrieval trial started with the presentation of a fixation cross for 2 s, followed by the presentation of the word-cue (the As or the Ds) for another 2 s. The first letter of the paired word (the probe) was then presented for 3 s and participants were asked to retrieve the target associate (half of the Bs and the Cs and all of the Es). The encoding context was not cued, mentioned, or in any other way made relevant in the retrieval task. At the end of each retrieval block, participants were then asked to retrieve the competitors (the remaining Cs and Bs). Participants responded orally, and their response was registered by the experimenter. To avoid muscle artifacts in the EEG data, participants were asked to withhold their response until the presentation of a question mark.

### E‌EG Recording and Preprocessing

The EEG was recorded continuously using a SynAmps RT Neuroscan amplifier (1 kHz sampling rate; left mastoid reference; bandwidth DC-3500 Hz; 24-bit resolution) from 62 active electrodes mounted in an elastic cap and positioned according to the extended 10–20 system. The EEG data were preprocessed using FieldTrip (Oostenveld et al. 2011) and in-house MATLAB scripts. Offline, the data were downsampled to 500 Hz and divided into two different epochs of interest: an encoding and a retrieval epoch. The encoding data were extracted from an epoch ranging from −1 to 7 s relative to the onset of the movie, to be used for the training of the pattern classifiers to distinguish the movie contexts. The retrieval epoch was created by selecting data ranging from −2 to 6 s relative to the onset of the word-cue. These data were used to test the neural pattern classifiers, that is, assessing reinstatement of the movie context, and to perform a standard univariate time–frequency analysis.

The epoched data were transformed to a linked-mastoid reference and baseline corrected (subtraction by the average amplitude of the epoch). Additionally, bipolar electrooculogram measures were computed using the FP1 electrode and an electrode placed below the left eye, and the FT9 and FT10 electrodes in the cap to respectively detect vertical (blinks) and horizontal eye movements, respectively. EEG epochs were physically inspected and those containing muscle or other artifacts, not related to blinks and horizontal eye movements, were manually removed. Independent component analysis was conducted and components representing oculomotor artifacts and muscle activity distinct from the EEG signal were removed. In addition, channels with consistent noise across participants were removed from the analysis (FT9/FT10), any remaining bad channels (if any) were interpolated, and the data were again visually inspected to remove any trials containing residual artifacts. The final analysis included an average of 34 AB trials (ranging between 30 and 36 trials), 34 AC trials (ranging between 29 and 36 trials), and 66 DE trials (ranging between 51 and 72 trials) per participant.

### Time–Frequency Decomposition

The signals from individual trials were transformed into time–frequency representations (TFRs). TFRs were obtained for frequencies ranging from 4 to 45 Hz, with a frequency step of 1 Hz, a time step of 0.05 s, and a wavelet width of 5 cycles, using the complex Morlet wavelet transform as implemented in FieldTrip. Brain oscillatory activity has been previously used to successfully train pattern classifiers to distinguish mnemonic representations (Jafarpour et al. 2014; Bramão and Johansson 2018) and is thought to support core mechanisms of episodic memory (Hanslmayr and Staudigl 2014; Hanslmayr et al. 2016; Schreiner and Staudigl 2020).

### Statistical Analysis and Multivariate Pattern Classification

We trained a classifier to discriminate patterns of brain activity associated with the context movies at encoding. MVPA was performed using a support vector machine (SVM), with a linear kernel, and a one-against-all strategy, as implemented in the MATLAB bioinformatics toolbox and following proposed protocols in the literature (Jafarpour et al. 2013). The pattern classifiers were trained on the averaged TFR over the course of each 6-s movie presentation during the encoding phase (Note: for periods in which movies were present up to 9 s, the first 6 s was used for analysis). The classifiers used the TFR from 60 channels, and thus the classifier was trained on 2520 possible features (60 channels ^*^ 42 frequencies). No additional baseline correction was performed on the TFR; instead the power at each frequency and channel was normalized across trials (Jafarpour et al. 2013). Classification was performed using the TFR signal from an average of 63 trials corresponding to the underwater context (range 53–71), 64 corresponding to the forest context (range 54–71), and 64 corresponding to the city context (range 54–72). We used a 10-fold cross-validation procedure to test the classifier at encoding. That is, the data were randomized and partitioned into 10 roughly equal-sized subsets, over which 10 training-test iterations were performed. Each partition was used as the test set once, with the remaining nine partitions used for training the classifier in that fold. In each cross-validation iteration, the model was used to predict the category of the left-out trials.

To visualize the contribution of each channel and frequency to classification performance, we reran the classification training in two analyses. The contribution of individual channels was assessed by a searchlight analysis in the spatial domain. The same classification procedure was repeated but including only the target channel and its adjacent neighbors (average seven, ranging between four and eight channels). Analogously, the contribution of each frequency was assessed by rerunning classification using the target frequency and adjacent frequencies (three frequencies except for the minimum and maximum frequency where only two frequencies were used). Classification performance was allocated to the target channel and to the target frequency and was contrasted against chance (33.3%). Classification accuracy that survived Bonferroni correction is reported (corrected *P*-value for frequencies: 0.05/42 comparisons = 0.0012; corrected *P*-value for channels: 0.05/60 comparisons = 0.0008).

The pattern classifier built and cross-validated with the encoding data was subsequently used to predict the target’s encoding context based on the retrieval data (i.e., the context of the to-be-retrieved target word) in trials for which participants showed successful target retrieval as well as for trials for which participants failed to retrieve the target. The testing was performed at 85 separate time bins, from −0.2 to 4 s relative to word-cue onset, with 0.05-s intervals, covering both the cue and the probe time window.

Each classification iteration produced a confusion matrix that summarizes the classification output. The rows of the matrices represent the true context categories whereas the columns represent the predicted context categories. The classification in the confusion matrix at a given site (*r*, *c*) expresses the number of observations having label *r* that the classifier labeled as *c*. Values on the diagonal of the matrix (*r* = *c*) correspond to the correct classifications where the true and the predicted categories are the same. As classification in the present study involved a balanced number of observations per category, we divided every element of the confusion matrix by the sum of its row so that every row sums to one. Classifier accuracy, defined as the percentage of classification attempts that correctly predicted the category of the observation, was computed as the mean of the diagonal of the confusion matrix averaged over cross-validation iterations and participants.

To determine the statistical significance of the observed classification accuracy, we generated the null distribution for each participant by conducting classifications with shuffled data. To test the significance of the classification at encoding we conducted 100 iterations and to test the statistical significance of the replay at retrieval we conducted 1000 iterations (to account for the increased number of data points at retrieval). At each iteration, the labels for the context movies were shuffled. Thus, each iteration yielded a distribution that contained no true information about the category of the movie but preserved overall smoothness and other statistical properties. To correct for multiple comparisons and test the statistical reliability of the classification obtained during encoding, we compared classification against the average classification obtained with the shuffled data. To correct for multiple comparisons and test the statistical reliability of the classification obtained during retrieval, we conducted a one-sample *t*-test for each of the 1000 iterations comparing classification performance against chance (33.3%). The distribution of the *t*-tests obtained with the shuffled data formed the nonparametric empirical null distribution, and the 97.5 and 2.5 percentiles of this distribution were used as the significance threshold for a two-tailed test, which corresponds to a significance threshold of 0.05. The classification performance at retrieval was smoothed using a moving average with a size of 0.1 s for display and for calculating the *t*-test of the nonparametric distribution. Classification accuracy was considered significant if the *t*-value obtained when comparing classification against chance (33.3%) was higher than the threshold *t*-value obtained in the permutation test (Osipova et al. 2006; Johnson et al. 2015).

Additionally, the results of this first permutation were corroborated with a Monte Carlo permutation test with a false discovery rate (FDR) correction as implemented in FieldTrip (Oostenveld et al. 2011). Here, the type-1 error was controlled by finding the FDR corrected *P*-value at each time point. A null distribution of the data was calculated by randomizing the data 1000 times across data points for each participant. At each permutation iteration, a *t*-test contrasting the classification in the shuffled data against chance (33.3%) was performed. FDR was used to estimate the probability of getting a false positive result given the observed positive results among the reference null distribution, considering a threshold of *P* < 0.05. Only results that were significant in both approaches are reported.

### Relationship Between Context Reinstatement and Memory Retrieval

To investigate the relationship between context reinstatement and episodic remembering, we investigated classification accuracy during retrieval as a function of memory performance. Classification accuracy for successful and unsuccessful retrieval trials was contrasted for the time intervals where significant replay was observed in the previous step. Critically, to avoid bias due to statistical nonindependence, we implemented a leave-one-subject-out (LOSO) cross-validation procedure (Esterman et al. 2010). This was done contrasting the classifiers, trained and tested on the data from all the other participants, against chance (33.3%) using a one-sample *t*-test and evaluating it with the thresholds of the obtained null distribution. This allowed us to identify, for each of the left-out participant, the time bins that showed classification different from chance over the course of the retrieval epoch. To select the specific time bins for each of the left-out participant, we identified the classifiers showing significant replay in a time interval within 0.2 s above and below the time window identified in the previous step. The identified time bins were used to extract the classification accuracy for successful and unsuccessful trials for the left-out participant. Outside these predefined time windows, we did not observe any other consistently significant classifier across iterations. Importantly, by using this procedure we avoided circularity in the analysis as independent data were used to select the time windows to contrast the memory replay in successful and unsuccessful trials.

For noncompetitive memory retrieval, a two-tailed paired sample *t*-test compared the mean classification accuracy for successful and unsuccessful retrieval trials. Additionally, for competitive retrieval, we further investigated the classifier evidence for target, competitor, and DE noncompetitive context as a function of memory performance. We entered the classifier accuracy, for the time bins where reliable replay was observed, in a repeated-measure analysis of variances (ANOVA) with the factors Memory Performance (successful vs. unsuccessful) and Context (target vs. competitor). The DE noncompetitive retrieval was excluded from the analysis to not violate the dependent-variable independence assumption of ANOVA. Only significant effects and interactions are reported. For these analyses, we only included participants with a sufficient number of trials per condition (>10 trials per condition).

### Statistical Analysis and Univariate Time–Frequency

A classical time–frequency univariate analysis was used to investigate episodic retrieval success effects. The power estimates at each time point were log-transformed and baseline corrected by the average power in a −1 to 0 s time window relative to the onset of the word-cue. The statistical significance of the effects was performed using a nonparametric cluster-based permutation test implemented in FieldTrip (Maris and Oostenveld 2007). This procedure, in a first step, performed dependent-sample *t*-tests to compare the conditions and identify statistically significant data samples (alpha = 0.05). All adjacent data samples (either spatial, temporal, or frequency neighbors) were then grouped into clusters, and the *t*-values within each cluster were summed and used to generate a cluster-level *t*-value. The type-1 error rate was controlled by evaluating the cluster-level test statistic under the randomization null distribution of the maximum cluster-level test statistic. This was obtained by randomizing the data between conditions for each participant. By creating a reference distribution from 10 000 random draws, the *P*-value was estimated according to the proportion of the randomization null distribution exceeding the observed maximum cluster-level test statistic (the so-called Monte Carlo *P*-value). In this way, significant clusters extending over time, frequency, and electrodes were identified.

To investigate episodic memory retrieval success effects, we contrasted successful with unsuccessful competitive and noncompetitive retrieval. This analysis was run on two different large time windows: the cue time window (0.3–1.5 s after cue onset) and the probe time window (0.3–1.5 s after probe onset) aiming to cover all the retrieval epoch, using the range of frequencies previously used in the classification analysis (4–45 Hz).

A separate analysis investigated if theta oscillatory activity tracked competitive retrieval. This analysis was motivated by previous work showing increased frontal theta activity, with an early onset, associated with memory interference (e.g., Hanslmayr et al. 2010; Bramão and Johansson 2017). The cluster-based permutation test was used to contrast competitive against noncompetitive retrieval in two different early time windows: the cue time window (0.1–0.6 s after stimulus onset) and the probe time window (2.1–2.6 s after stimulus onset) and using a range of frequencies in the theta band (3–6 Hz).

## Results

### Behavioral Results

For each participant and condition, cued-recall accuracy, defined as the percentage of correct responses, was quantified. The behavioral impact of memory competition on target memory retrieval was investigated as a function of word-pair type (AB vs. AC vs. DE word-pairs) with a repeated-measure ANOVA. Results revealed a significant effect of word-pair [*F*(2, 58) = 6.71, *P* = 0.002, ${\eta}_P^2$ = 0.19; see Fig. 1*C*]. Planned pairwise comparisons showed evidence for proactive, but not retroactive, interference. That is, memory performance for AC targets was significantly worse when compared with both DE noncompetitive retrieval [*t*(29) = −3.66, *P* = 0.001, *d* = −0.67] and AB target competitive retrieval [*t*(29) = −2.18, *P* = 0.037, *d* = −0.40]. By contrast, AB and DE retrieval did not differ [*t*(29) = −1.09, *P* = 0.28, *d* = −0.20] (Fig. 1*C*).

Further analyses showed that the behavioral interference effect was neither affected by the encoding block type nor by output interference (see Supplementary Note 1). Additionally, the interference effect remained after matching the DE control word-pairs by the serial position of the AB and AC word-pairs (see Supplementary Note 2). Finally, at the end of each block, after the retrieval of the targets (either the B or the C), participants also were asked to retrieve the competitors (either the C or the B). In this final test, memory performance for AC competitors and targets was lower than for AB competitors and targets; however, no significant interaction between word-pair (AB vs. AC word-pairs) and item status (target vs. competitor) was observed (see Supplementary Note 3).

### Brain Activation Patterns Related to the Encoding Context

To quantify context reinstatement during retrieval, we first trained a neural pattern classifier to distinguish the oscillatory brain activity (across channels and spanning 4–45 Hz) associated with the context movie during encoding (Fig. 1*B*,*D*). Notably, classification accuracy was significantly above chance for all movies [underwater: *t*(29) = 8.19, *P* < 0.001, *d* = 1.50, threshold *t*-value = 1.55; forest: *t*(29) = 2.73, *P* = 0.011, *d* = 0.50, threshold *t*-value = 2.09; city: *t*(29) = 6.72, *P* < 0.001, *d* = 1.23, threshold *t*-value = 2.21] and for the mean across movies [*t*(29) = 8.38, *P* < 0.001, *d* = 1.53, threshold *t*-value = 1.84]. A repeated-measure ANOVA with the factor Movie (underwater vs. forest vs. city) revealed that decoding accuracy of encoding context differed [*F*(2, 58) = 8.90, *P* < 0.001, ${\eta}_P^2$= 0.24]: classification was lower for forest compared with both underwater [*t*(29) = −2.79, *P* = 0.007, *d* = −0.48] and city [*t*(29) = −4.14, *P* = 0.001, *d* = −0.84] but comparable for underwater and city [*t*(29) = −1.35, *P* = 0.18, *d* = −0.24]. Importantly, because the movie contexts were counterbalanced within participants and assigned to all conditions across blocks, any difference in decoding accuracy between encoding contexts cannot explain differences between the retrieval conditions (AB, AC, DE).

To visualize which features contributed most to classification, we investigated the contribution of each feature (i.e., channels and frequencies) (see Materials and Methods for details; see Fig. 1*E*). This analysis revealed that oscillatory activity (4–20 Hz) recorded at posterior electrode channels contributed the most to accuracy, suggesting that classification largely depends on visual processing that distinguishes the three movie contexts. This result replicates and extends previous findings obtained with static visual stimuli (Jafarpour et al. 2014; Kaneshiro et al. 2015; Kurth-Nelson et al. 2015; Bramão and Johansson 2018).

### Context Reinstatement During Noncompetitive Target Retrieval

Using the classifier built on the encoding data, we next investigated incidental context reinstatement during DE noncompetitive retrieval. This is an important step to validate our methodological approach and to replicate and extend previous findings in the literature (e.g., Manning et al. 2011; Diana et al. 2013; Gershman et al. 2013; Folkerts et al. 2018). Given the contextual nature of episodic memory retrieval, noncompetitive target retrieval should be accompanied by the reinstatement of the encoding context already during the initial cue time window. As the word-cue was previously encountered in a single encoding event, it could readily prompt retrieval of the sought-after memory trace (Fig. 1*A*,*F*). The neural pattern classifier, trained during encoding, was applied over the time course of DE noncompetitive retrieval. As no movie context was presented during retrieval, any classification evidence reflects the replay of the neural patterns diagnostic of the encoding context. As we would expect stronger context reinstatement for successful target retrieval (e.g., Herweg, Sharan, et al. 2020a), this analysis was first restricted to trials in which the participants remembered the target word. Moreover, to investigate context reinstatement as a function of memory performance, we then contrasted classifier accuracy for successful and unsuccessful target retrieval, in the time bins for which classifier accuracy differed from chance. Only participants (*n* = 27) with enough trials (>10 trials) in both conditions were considered. To avoid circularity in the data, we implemented a LOSO cross-validation procedure to identify the time windows for each participant (Esterman et al. 2010).

**Figure 2:**
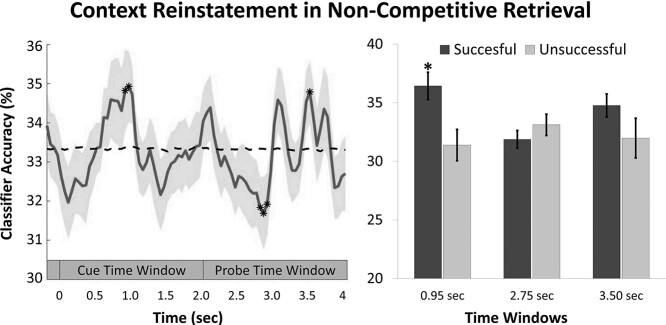
On the left is shown the averaged classification accuracy for successful DE noncompetitive retrieval in relation to the target context. Chance level is shown in dashed line. For illustration purposes, classification was smoothed using a moving average with a size of 0.1 s. On the right is shown the averaged classifier accuracy for noncompetitive retrieval as a function of retrieval success versus failure in the time-bins where reliable neural reinstatement was observed for successful trials. Highlighted (^*^) are the time-bins for which classification performance was significantly different from chance. Error bars represent the standard error (SE) of the mean.

Figure 2 shows the temporal dynamics of incidental target context reinstatement for DE noncompetitive retrieval. As predicted, we observed that the neural pattern that corresponded to the encoding context of the target was reinstated already in the cue time window, starting at 0.9 s post cue onset [mean ± SD at 0.95 sec = 36.4 ± 4.3, *t*(29) = 2.70, *P* = 0.01, *d* = 0.49, threshold *t*-value = 2.29]. Critically, this context reinstatement was significantly stronger for successful compared with unsuccessful target retrieval [*t*(26) = 2.42, *P* = 0.023, *d* = 0.46]. Furthermore, a one-sample *t*-test confirmed that classification accuracy for successful trials was significantly above chance [*t*(29) = 2.63, *P* = 0.013, *d* = 0.48] whereas it was not for unsuccessful trials [*t*(26) = −1.40; *P* = 0.17, *d* = −0.27] (Fig. 2). Moreover, classifier evidence for target context reinstatement in the cue time window was stronger for noncompetitive retrieval compared with competitive retrieval (see Supplementary Note 4).

Classification performance was significantly different from chance later in the probe time window, between 2.7 and 2.9 s [mean ± SD at 2.75 s = 31.4 ± 3.8, *t*(29) = −2.17, *P* = 0.038, *d* = −0.40, threshold *t*-value = −2.01] and at 3.5 s after trial onset [mean ± SD = 35.6 ± 3.6, *t*(29) = 2.30, *P* = 0.029, *d* = 0.42, threshold *t*-value = 2.12]. However, classification performance was comparable for successful and unsuccessful retrieval in these later time windows (all *P*s > 0.2) (Fig. 2). The tendency observed in the data for a negative classification between 2.7 and 2.9 s may indicate that a context other than the DE was being reinstated during this time period. Although there was no systematic bias toward one of the other two contexts (Supplementary Note 5), it is interesting to note the numeric tendency for participants to preferentially reinstate AB and AC contexts compared with DE contexts when the DE target was successfully retrieved. This can potentially be explained by participants engaging in post-retrieval monitoring processes, such as mentally imagining the retrieved word as encountered in any of the alternative movie contexts. Furthermore, in the end of the retrieval period, there is a tendency for context reinstatement of the DE target, thus suggesting the retrieval of contextual information with or without access to the discrete target event.

Taken together, these results suggest that target retrieval is accompanied by the reinstatement of the original encoding context and more so during successful remembering. These results extend previous findings to a novel paradigm in which the encoding context is completely incidental to the memory task and participants are only asked to retrieve the embedded discrete target words.

**Figure 3:**
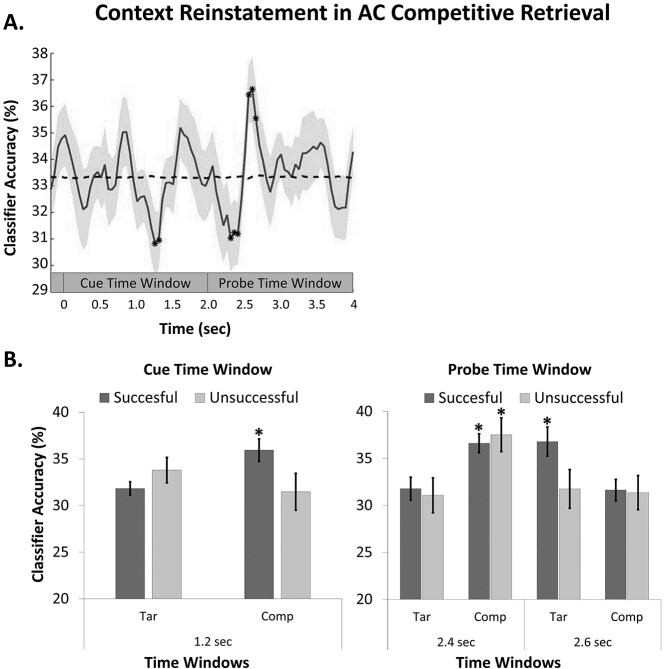
(*A*) averaged classification accuracy for AC retrieval in relation to target context movie. Chance level is shown in dashed line. For illustration purposes, the classification was smoothed using a moving average with a size of 0.1 s. (*B*) Classifier evidence for target and competitor context as a function of retrieval success is shown. The time-bins depicted correspond to the ones where reliable neural reinstatement was observed for successful trials. Highlighted classification evidence (^*^) indicates that classification accuracy was significantly different from chance. Error bars represent the standard error (SE) of the mean.

### Context Reinstatement During Competitive Retrieval

The previous analysis confirmed that encoding context is incidentally reinstated in our novel paradigm and covaries with successful target retrieval on noncompetitive (DE) trials. Next, we investigated such incidental context reinstatement during competitive retrieval. During the word-cue time window, participants were presented with a cue (i.e., A) associated with multiple traces (i.e., B and C). We reasoned that if the encoding contexts specifying the original events are incidentally reinstated during retrieval competition, we would observe reduced classifier accuracy for the target encoding context in this early time window. In the ensuing probe time window, participants were presented with a specified test probe (first letter of target word) that indicated the memory trace to be retrieved (i.e., either the B or the C), allowing for interference resolution and selective retrieval of the target memory. We predicted that such resolution would be accompanied by the emergence of target context reactivation as reflected in increasing classifier accuracy (Fig. 1*A*,*F*). To test these predictions, the neural pattern classifier, trained during encoding, was applied over the time course of competitive retrieval. As our behavioral data showed proactive, but not retroactive, interference, we examined successful AB and AC competitive retrieval separately.

**Figure 4:**
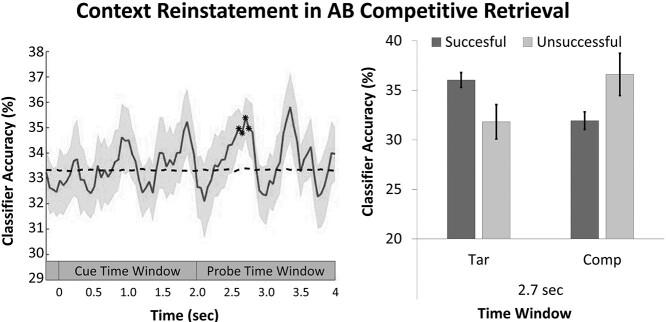
On the left is shown the averaged classification accuracy for AB retrieval calculated in relation to target context movie. Chance level is shown in dashed line. For illustration purposes, the classification was smoothed using a moving average with a size of 0.1 s. On the right the classifier evidence for target and competitor context as a function of retrieval success is shown. The time-bins depicted correspond to the ones where reliable neural reinstatement was observed for successful trials. Highlighted classification evidence (^*^) indicates that classification accuracy was significantly different from chance. Error bars represent the standard error (SE) of the mean.

The temporal dynamics of the incidental reinstatement of target context are shown in Figure 3 for AC competitive retrieval and in Figure 4 for AB competitive retrieval. As predicted, the neural reactivation of the target context emerged only in the late probe time window (between 2.5 and 2.8 s) for both AC and AB retrieval, approximately 0.5–0.8 s after probe onset [AC word-pairs: mean ± SD at 0.55 s = 37.8 ± 7.4, *t*(27) = 3.16, *P* = 0.0038, *d* = 0.60, threshold *t*-value = 2.13; AB word-pairs: mean ± SD at 0.75 s = 36.5 ± 6.3, *t*(29) = 2.76, *P* = 0.009, *d* = 0.51, threshold *t*-value = 2.14]. Supplementary Note 4 shows that classifier evidence for target context reinstatement in the probe time window (~0.5–0.8 s after probe onset) was stronger for competitive retrieval than for DE noncompetitive retrieval.

AC competitive retrieval—the condition associated with proactive interference—showed classification performance significantly below chance in two additional time windows (Fig. 3). First, early in the word-cue time window, at 1.25 s after trial onset (mean ± SD = 30.3 ± 7.0, *t*(27) = −2.14, *P* = 0.041, *d* = −0.41, threshold *t*-value = −1.95] and next, in the probe time window, between 2.35 and 2.45 s [mean ± SD at 0.45 s = 30.8 ± 6.9, *t*(27) = −1.86, *d* = −0.35, threshold *t*-value = −1.85]. Critically, negative classification indicates that a context other than the target context was reinstated. In the following analyses, we investigate if this finding is associated with the reactivation of the brain patterns associated with the context of the competing memory.

### Context Reinstatement Tracks Retrieval Competition During AC Retrieval

Having identified the time bins for which classifier accuracy was reliably different from both chance and noncompetitive retrieval, we next investigated classifier accuracy as a function of interference resolution. As we only observed reliable proactive interference, we restricted the analysis to AC competitive retrieval trials. To avoid statistical circularity, we implemented a LOSO cross-validation procedure to identify the analytic time windows for each specific participant (Esterman et al. 2010). The analysis was run using a subsample of participants (*n* = 25 for AC retrieval) with a sufficient number of trials (>10).

The temporal dynamics of competition and interference resolution were examined in the probe time window. In this time window, participants were presented with a first-letter probe that indicated which of the two associates that was goal relevant (either the B or the C). Thus, it is during this time window we would expect interference control mechanisms to operate in the service of selective retrieval. As reported above, analyses showed 1) reliable target context reinstatement for AC retrieval during this time window (between 2.5 and 2.8 s) and 2) preceding evidence of below-chance target classification (between 2.35 and 2.45 s), conceivably due to an incidental context reinstatement of the competing AB memory. To directly evaluate this interpretation, classifier accuracy for AC word-pairs in the probe time window was investigated with a three-way repeated-measure ANOVA with the factors Time Window (early vs. late), Memory Performance (successful vs. unsuccessful), and Context (target vs. competitor). The DE noncompetitive condition was excluded from the analysis to avoid violating the assumption of independence among the dependent variables. The ANOVA showed a significant interaction between Time Window and Context [*F*(1, 23) = 28.81, *P* < 0.001, ${\eta}_P^2$= 0.56]. Additionally, the interaction between Time Window and Memory Performance [*F*(1, 23) = 9.03, *P* = 0.006, ${\eta}_P^2$= 0.28] was significant (Fig. 3). Corroborating the idea that interference resolution is paralleled by the incidental reinstatement of the target context, we verified that, in the late time window, evidence for the target context was stronger compared with the competitor context but only when competition was resolved [successful retrieval: *t*(27) = 2.22, *P* = 0.035, *d* = 0.42; unsuccessful retrieval: *t*(24) = −0.11, *P* = 0.91, *d* = −0.02]. Moreover, classifier evidence for the target context was stronger in the late compared with the early time window but again only for successfully resolved competition [Successful retrieval: *t*(27) = 2.97, *P* = 0.006, *d* = 0.56; unsuccessful retrieval: *t*(24) = 0.54, *P* = 0.60, *d* = 0.11].

Consistent with the idea that successful competition resolution was preceded by the incidental reinstatement of the competing context, we further observed that, in the early probe time window, the reactivation of the competitor context was stronger than the reactivation of target context [successful retrieval: *t*(27) = 2.54, *P* = 0.017, *d* = 0.48]. This tendency was also present for unsuccessfully resolved competition [unsuccessful retrieval: *t*(24) = 1.83, *P* = 0.08, *d* = 0.37]. Evidence for the competitor context was also stronger in the early than in the late time window, irrespective of retrieval success [Successful retrieval: *t*(27) = 4.24, *P* < 0.001, *d* = 0.80; unsuccessful retrieval: *t*(24) = 2.99, *P* = 0.007, *d* = 0.61]. Finally, classifier evidence for the competitor context, in the early probe time window, was significantly above chance (33.3%) both for successful [*t*(27) = 3.21, *P* = 0.003, *d* = 0.61] and unsuccessful [*t*(24) = 2.30, *P* = 0.030, *d* = 0.46] retrieval whereas classifier evidence for target context was only significantly different from chance in the late time window and for successful target retrieval [*t*(27) = 2.21, *P* = 0.035, *d* = 0.42].

These novel findings show that the encoding context is incidentally reinstated during competitive retrieval and that such reinstatement tracks retrieval competition and interference resolution. Proactive interference was neurally associated with the incidental reactivation of the encoding context of the competing memory trace. Competitor reinstatement was observed very early, approximately 350–450 ms after probe onset (i.e., 2.3 s after trial onset). Competition resolution followed immediately thereafter, that is, approximately 550–800 ms post probe presentation (i.e., 2.5 s after trial onset) as reflected in the cortical reinstatement of the encoding context of the target.

Next, we turn to the cue time window. Surprisingly, we observed that AC retrieval was associated with classifier evidence for below-chance target context reinstatement already in the cue time window (1.25 s after trail onset) (Fig. 3). In this time window, participants were provided with a word-cue (i.e., the A) pointing at multiple memory traces (i.e., B and C) without specification of the goal-relevant memory. To further understand this effect, we investigated the possibility that the word-cue presentation gives rise to incidental reinstatement of the context associated with the strongest memory trace; that is, the competing AB encoding context. This prediction was tested by examining classifier accuracy for AC word-pairs, during the word-cue time window, with a repeated-measure ANOVA with the factors Memory Performance (successful vs. unsuccessful) and Context (target vs. competitor). The analysis revealed no significant effects (all *P*s > 0.09). Nonetheless, the notion that proactive interference may be driven by competitor reactivation already in the word-cue time window is supported when focusing on the successful retrieval trials. Paired-sample *t*-tests showed stronger classification evidence for the AB competitor context than for the AC target context [*t*(27) = 2.32, *P* = 0.028, *d* = 0.44], and above-chance evidence for AB competitor context reinstatement [*t*(27) = 2.14, *P* = 0.042, *d* = 0.40; Fig. 3]. Thus, it seems that AB competitor context reactivation occurs early during the trial, already in the cue time window, and its reactivation dominates retrieval until the probe is presented. At this moment, interference resolution takes place and AC target context reinstatement is observed.

The tendency to observe greater classifier evidence for the competitor, in a time window in which participants did not know which was the relevant memory trace for the trial, may seem counterintuitive. Notice, however, that this was only the case for AC word-pairs. To give further insight into this finding, we ran a univariate analysis, contrasting AC and AB word-pairs versus DE word-pairs in the theta band (3–6 Hz), in the cue and probe time windows. Previous studies have indicated that memory interference is associated with increased frontal theta activity (e.g., Hanslmayr et al. 2010; Staudigl et al. 2010; Waldhauser et al. 2012; Bramão and Johansson 2017). This analysis showed an increased early theta activity in the word-cue time window for AC but not for AB word-pairs (see Supplementary Note 6). This result aligns well with our behavioral findings. We only observed proactive interference when participants were asked to retrieve the C targets in the AC word-pairs. This indicates that our paradigm instigated stronger AB associations compared with AC associations. Therefore, when participants were given the word-cue A, the AB association may be the most readily reactivated event. A challenge to this idea, though, is that we did not observe classification evidence for the target context during AB retrieval in this cue time window. However, an exploratory analysis of AB retrieval as function of encoding block type (i.e., order of AB/AC/DE) did reveal target context reinstatement for the block type that promotes AB memory (see Supplementary Note 7).

### Context Reinstatement During AB Retrieval

Even though there was no evidence of retroactive interference during AB retrieval, for completeness, we investigated if classifier evidence for target context is also evident during AB retrieval trials as a function of retrieval success. Successful retrieval of AB word-pairs was associated with above-chance target context classifier accuracy in the probe time window (between 2.5 and 2.8 s) (Fig. 4). This analysis was restricted to a subsample of participants (*n* = 24 for AB retrieval with >10 trials). We again avoided statistical circularity by defining participant-specific analytic windows using a LOSO approach. Classifier evidence for AB word-pairs was investigated with a two-way repeated-measure ANOVA with the factors Memory Performance (successful vs. unsuccessful) and Context (target vs. competitor). The ANOVA showed a marginal interaction between the two factors [*F*(1, 24) = 3.64, *P* = 0.069, ${\eta}_P^2$ = 0.13]. Planned pair-wise comparisons revealed that classifier evidence for the target context tended to be stronger for successful compared with unsuccessful AB retrieval [*t*(24) = 1.99, *P* = 0.059, *d* = 0.40]. Additionally, classifier evidence for the target context was higher than evidence for the competitor context when memory performance was successful [*t*(29) = 2.8, *P* = 0.009, *d* = 0.51]. Furthermore, classifier accuracy for the target context was significantly above chance (33.3%) for successful retrieval trials [*t*(29) = 3.6, *P* = 0.001, *d* = 0.65]) but not for unsuccessful trials [*t*(23) = −0.84, *P* = 0.14, *d* = −0.30] (Fig. 4).

In sum, successful AB target retrieval was accompanied by the reactivation of the encoding movie context but only when memory retrieval was successful.

### Context Reinstatement As a Function of Memory for the AB/AC Triplet

We next sought to determine how modulations in context reactivation, as retrieval unfolds, relates to the long-term accessibility of the two discrete events, that is, the target and the other associate. Interestingly, classification accuracy reported in Figures 2–4 shows a pattern going above and below chance. This pattern may indicate that participants are alternating between two associated memory representations. We tested this idea by investigating the presence of reliable above- and below-chance classification in the retrieval competition condition. We examined whether classifier evidence was predictive of remembering AB and AC associates. To have enough trials, AB and AC retrieval conditions were collapsed and retrieval trials were separated into three categories: 1) remembering both the target and the associate; 2) retrieving the target memory but failing to retrieve the associate; and 3) failing to retrieve the target but remembering the associate. Supplementary Note 8 shows that remembering the associated memory trace was accompanied by reinstatement of the associate’s context in the cue time window. In contrast, target memory retrieval was accompanied by the reinstatement of the target’s context in the probe time window. Importantly, classification above and below chance was only observed when participants remembered both memories, which indirectly suggests that the pattern reported in Figures 3 and 4 may be related to the maintenance of and alternating between the two associated memory traces.

### Time–Frequency Representations of Successful Target Retrieval

The previous analysis investigated the temporal dynamics of context reinstatement in noncompetitive and competitive retrieval. Next, we sought to examine the neural correlates of target-word retrieval during competitive and noncompetitive retrieval. A univariate analysis was run contrasting successful and unsuccessful retrieval, with a cluster-based permutation test (Maris and Oostenveld 2007), over the time course of retrieval and in the same frequency range used in the MVPA.

**Figure 5:**
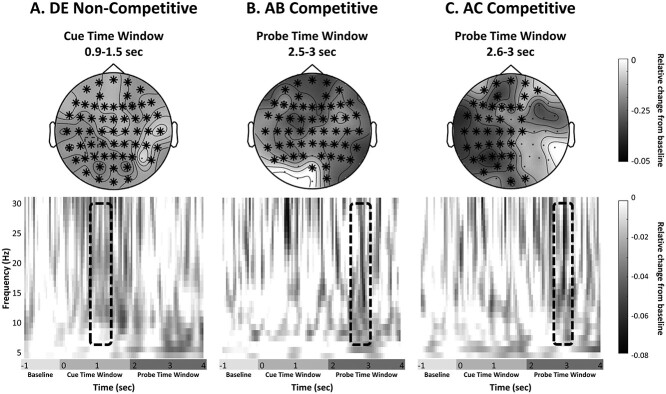
Data shown for retrieval effects (successful vs. unsuccessful retrieval). (*A*) DE noncompetitive retrieval, (*B*) AB competitive retrieval, and (*C*) AC competitive retrieval. The upper row shows the topography of the effects. Electrodes that reached significance are highlighted (^*^). The lower row shows the averaged time–frequency representations from a representative channel (C5). The area showing significant effects is highlighted.

Critically, we observed significant effects in the alpha/beta frequency range in time windows that overlap with the ones in which the pattern classifiers identified reliable incidental reinstatement of target context. DE noncompetitive retrieval was associated with increased alpha/beta desynchronization present in the cue time window, between 0.9 and 1.5 s (*P* = 0.02, *d* = −0.57, see Fig. 5*A*). Competitive retrieval, in contrast, showed significant retrieval success effects, characterized by increased desynchronization in the alpha/beta band, not until the later probe time window, between 2.5 and 3 s (AB retrieval: *P* = 0.02, *d* = −0.53; AC retrieval *P* = 0.04, *d* = −0.16, see Fig. 5*B*,*C*).

Interestingly, the temporal dynamics of these data overlap with the results from the multivariate approach, showing that successful target retrieval occurs in the same time window as context target reinstatement. Moreover, the current results support recent accounts highlighting the role of alpha/beta desynchronization in episodic memory retrieval (Hanslmayr et al. 2012; Hanslmayr et al. 2016; Griffiths et al. 2021) and corroborate previous findings in the literature (Manning et al. 2011; Jafarpour et al. 2014; Staudigl et al. 2015; Michelmann et al. 2016; Waldhauser et al. 2016).

## Discussion

The ability to remember an episode from our personal past is often hindered by competition arising from similar overlapping events. Here, we investigated whether memory competition is characterized by the incidental reinstatement of the incidental contexts in which discrete but overlapping events were embedded during encoding. Context reinstatement during competitive retrieval may reduce the need for cognitive control to minimize the effects of memory interference. By using MVPA of high temporal-resolution EEG data, we examined target and competitor context reinstatement over the time course of memory retrieval. We provide evidence showing that memory competition is indeed associated with the simultaneous and incidental reactivation of contextual details associated with both target and competing memories. Importantly, this incidental context reinstatement tracks retrieval competition and interference resolution and offers new insights into the temporal dynamics of selective memory retrieval.

We successfully trained a pattern classifier to distinguish the oscillatory brain activity induced by dynamic movie contexts during encoding (Fig. 1*C*,*D*). The classifier was then applied over the time course of retrieval to monitor the incidental reinstatement of the context. Our results show that noncompetitive retrieval was associated with the reinstatement of the target memory’s encoding context between 0.9 and 1 s after word-cue onset. Critically, such context reactivation predicted successful memory performance, thus excluding the possibility that our results were solely driven by visual imagery and other nonmnemonic strategies (Fig. 2). Note that context was completely incidental to the retrieval task (Smith and Vela 2001; Stark et al. 2018). Thus, this effect likely reflects the incidental reinstatement of the encoding context rather than its intentional retrieval. Moreover, our data show that the timing of context reinstatement overlaps with the timing of successful retrieval of the target word (Fig. 5*A*). This replicates previous findings (e.g., Manning et al. 2011; Diana et al. 2013; Gershman et al. 2013; Miller et al. 2013; Staudigl et al. 2015; Folkerts et al. 2018; Herweg, Sharan, et al. 2020a) indicating that successful target retrieval is accompanied by a simultaneous accessibility of the encoding context. Importantly, the incidental nature of context in our paradigm aligns well with the idea that the hippocampus binds together the slowly drifting contextual changes with more discrete elements of an event (Polyn et al. 2009; Howard 2017; Yonelinas et al. 2019; Kahana 2020), which consequently allows the concurrent recollection of all its elements via pattern completion (Horner et al. 2015; Ritchey and Cooper 2020).

Having established a way to quantify incidental reinstatement of encoding context, our primary aim was to investigate if it tracks competition between related events encoded in different contexts and successful interference resolution. Behaviorally, we observed reliable proactive interference. Neurally, we observed that the encoding context, in which overlapping, competing events were embedded during encoding, was incidentally reinstated during competitive retrieval. Proactive interference was associated with the reactivation of the competing movie context, occurring early during retrieval, likely existing in the cue time window, and spreading into the early portion of the probe time window (Fig. 3*A*). Critically, this was the case for both resolved and unresolved proactive interference (Fig. 3*B*), indicating that competitive retrieval is characterized not only by the reactivation of the competing item but also of encompassing information, including the contextual details specifying its encoding context. When participants successfully resolved memory interference, competitor context reinstatement was followed by target context reactivation. Our data show that successful competition resolution was associated with target context reinstatement and that such reinstatement predicted interference resolution. This finding is consistent with the idea that incidental reinstatement of the contextual features, specifying the encoding episode, may aid during competitive retrieval. Access to the encoding context could facilitate goal-relevant retrieval or post-retrieval target feature selection by differentiating overlapping events and reducing memory interference.

One of the most prominent accounts of competitive retrieval posits that cognitive control mechanisms actively inhibit competing memories that are coactivated with the target memories (Levy and Anderson 2002; Anderson 2003). A consequence of such inhibitory mechanism is later forgetting of the competitors (Anderson et al. 1994). The incidental reinstatement of the contextual features during competitive retrieval may be an alternative/complementary mechanism of handling memory interference. In fact, a recent study shows that strategically adopting a retrieval orientation toward items encoded in a particular context reduces memory interference and, as a corollary, protects related and otherwise competing memories from inhibition and later forgetting (Kerrén et al. 2021).

Our data show that target context reinstatement, predictive of interference resolution, was observed early at approximately 0.50 s after first-letter probe onset (that is, ~2.5 s after trial onset). Interestingly, the timing of this effect overlapped with the timing of successful target word retrieval (Figs 3 and 5), indicating that encoding context and target word are coactivated in a parallel fashion during competition resolution. Previous studies have shown that the cortical reinstatement leading to memory retrieval can occur as early as approximately 0.5 s after cue onset (Jafarpour et al. 2014; Kurth-Nelson et al. 2015; Bramão and Johansson 2018; Staresina and Wimber 2019). Our data show that interference resolution may be remarkably fast as the cortical reinstatement of the target context showed a similar temporal profile despite the preceding retrieval competition. This corroborates the idea that mechanisms handling retrieval competition operate very early and swiftly to pave the way for goal-relevant target reactivation and/or selection (Waldhauser et al. 2012).

Interestingly, while target context reactivation associated with interference resolution was observed approximately 0.50 s after probe onset, target context reactivation for noncompetitive retrieval occurred later, approximately 0.95 s after word-cue onset. Hippocampal pattern completion leading to cortical reinstatement may depend on multiple factors, such as memory strength and goal-directed biasing. In fact, some studies have shown that the early memory signals initiated by the bottom-up hippocampal–cortical reinstatement are sent to posterior parietal cortical regions where cortical reinstatement is further refined or integrated in a top-down goal-directed fashion (Wagner et al. 2005; Bergström et al. 2013; Kuhl et al. 2013; Favila et al. 2018; Staresina and Wimber 2019). This suggests that the timing of cortical reinstatement leading to successful memory retrieval may occur at different times depending on the specific requirements of the memory task.

The classification results here reported (Figs 2–4) show systematic fluctuations above and below chance level for target and competing context reinstatement, respectively. When participants only remembered one of the word-pair associates no such pattern was observed (Supplementary Note 7). It is conceivable that this classification above and below chance reflect an alternating between the two possible memory traces. Previous studies have linked classification accuracy oscillating at delta frequencies with switching between representational states in working memory (e.g., de Vries et al. 2019; de Vries et al. 2020). Future studies are needed to provide further insight into how these mechanisms operate in situations of episodic memory retrieval.

Our data show proactive but not retroactive interference; that is, retrieval competition was only present when participants were asked to retrieve the C targets in the AC word-pairs. This indicates that our paradigm may have instigated stronger AB compared with AC associations. In fact, during AC retrieval, we observed a tendency for classifier evidence for competitor (AB context) to be stronger than for the target (AC context), existing in the cue time window, and when participants did not know which of the memory traces (either B or C) was goal relevant. During the encoding task, participants were asked to imagine the word-pairs in the context movies. This may have promoted a strong memory representation of the AB association that was difficult to disrupt/update when participants were next asked to create an AC association. Therefore, when participants were given the word-cue A, the competing memory B was reinstated, causing proactive interference. Additionally, target reactivation for the AB word-pairs was observed when AB and AC were presented further apart during the encoding block. Future studies should further explore the mechanisms mediating retroactive and proactive interference. It may be that these two types of interference are mediated by at least partially distinct neural mechanisms operating at different phases (encoding vs retrieval) of memory. For instance, extant data indicate that memory reactivation of AB during later AC learning predicts resistance to retroactive interference (Kuhl et al. 2010, 2011; Koen and Rugg 2018) and the present data (along with Kuhl et al. 2011) indicate that proactive interference emerges, in part, from AB reactivation during AC retrieval. However, extant data diverge on whether proactive interference is partially explained by AB reactivation during AC learning (Koen and Rugg 2018), with across-study variability perhaps relating to the extent to which overlapping events are integrated in memory (Shohamy and Wagner 2008; Schlichting and Preston 2015; Favila et al. 2018; Chanales et al. 2019).

Strikingly, in the cue time window, our data show competitor context reactivation for successful but not for unsuccessful memory retrieval (Fig. 3). This observation suggests that forgetting is not entirely attributable to mnemonic interference arising when a competing memory impairs target retrieval (Levy and Anderson 2002; Anderson 2003). Indeed, recent work demonstrates that the reactivation of overlapping competing memories does not necessarily come with a cost but may benefit memory by promoting integration of the overlapping memories (e.g., Chanales et al. 2019). Future studies are needed to investigate the tradeoff between memory interference and integration; recent studies suggest that context may play an important role moderating this interaction (Libby et al. 2019; Cox et al. 2021).

Altogether, our results provide novel electrophysiological evidence that episodic recollection involves the incidental reinstatement of the contextual details of past events during competitive retrieval. Specifically, we show that proactive interference resolution is seen in the eventual reinstatement of the target context and that memory interference is characterized by an early and incidental reinstatement of the competitor context. Episodic memory is, by definition, context dependent (e.g., Estes 1955; Polyn and Kahana 2008; Polyn et al. 2009; Howard 2017). Here we show, for the first time, that retrieval competition is associated with the incidental reinstatement of the episodic contexts in which the competing items were encoded. Future studies could investigate the functional role of such reinstatement, possibly contributing to the separation of highly overlapping episodes in order to reduce and resolve memory interference—mechanisms that are fundamental to selectively remembering our personal past.

## Notes

The authors thank all volunteers who participated in this study. The authors would like to thank Eysteinn Ívarsson and Tim Larsson for their assistance during stimuli preparation and data acquisition.*Conflict of Interest:* None declared.

## Funding

Swedish Research Council (VR 2015-01180); and the Marcus and Amalia Wallenberg Foundation (MAW 2015.0043).

## Supplementary Material

CerebralCortex_Supplementary_Material_IB_JJ_AW_MJ_R1_bhab529
