# Supplementary material for: Encoding contexts are incidentally reinstated during competitive retrieval and track the temporal dynamics of memory interference
Source: Cereb Cortex. 2022 Feb 1;32(22):5020–35. doi: 10.1093/cercor/bhab529 (PMC9667177; doi:10.1093/cercor/bhab529)
Supplement: CerebralCortex_Supplementary_Material_IB_JJ_AW_MJ_R1_bhab529 [file cerebralcortex_supplementary_material_ib_jj_aw_mj_r1_bhab529.zip › CerebralCortex_Supplementary_Material_IB_JJ_AW_MJ_R1_bhab529.docx]

**Supplementary Material**

**Supplementary Note 1: The behavioral interference effect is neither affected by the encoding block type nor by output interference**

We investigated if the block type and the recall order moderated the observed interference effect. DE word-pairs were presented either in the beginning of the block (DE, AB and AC), in the middle (AB, DE and AC) or in the end of the block (AB, AC and DE). Target memory retrieval was investigated in a repeated-measures ANOVA with the factors, Word-Pair (AB vs. AC vs. DE) and Block Type (DE beginning vs. DE middle vs. DE end). Neither the effect of Block Type nor the interaction between Block Type and Word-Pair was significant (all *p*s > 0.4), showing that the interference effect was comparable across block types.

Next, we investigated if the interference effect was modulated by the recall order at retrieval. In a single block, we tested memory performance for eight targets in a random order (4 DE targets, 2 AB targets, and 2 AC targets). A repeated measures ANOVA with the factors Word-Pair (AB vs. AC vs. DE) and Retrieval Order (1&2 vs. 3&4 vs. 5&6 vs. 7&8 position) was used to investigate if target memory was affected by output interference. Neither the effect of Retrieval Order nor the interaction between the two factors was significant (all *p*s > 0.3), showing that the interference effect here observed cannot be explained by output interference.

**Supplementary Note 2: The interference effect is not affected by differences in the serial position of AB/AC and DE word-pairs**

We investigated if the observed interference effect remained after matching the DE control word-pairs by the serial position of the AB and AC word-pairs. AB word-pairs were always presented after AC word-pairs. DE word-pairs, in contrast, were presented in the beginning, in the middle and in the end of the block. We therefore decided to examine the interference effect in blocks with matched serial position between the control DE word-pairs and the AB/AC word-pairs. The retroactive interference was investigated using data only from blocks where DE appeared in the beginning and in the middle, and proactive interference where DE appeared in the middle and in the end of the block. Memory performance for AB and AC word-pairs was contrasted with memory performance for DE word-pairs. We observed a significant proactive interference [DE word-pairs = 0.71 ± 0.20; AC word-pairs = 0.63 ± 0.22; *t*(29) = -2.91, *p* = 0.007, *d* = -0.53], but no retroactive interference [DE word-pairs = 0.71 ± 0.21; AB word-pairs = 0.68 ± 0.20; *t*(29) = -1.43, *p* = 0.16, *d* = -0.26]. This analysis replicated the previous one and shows that serial position does not interact with the interference effect.

**Supplementary Note 3: Competitor accessibility is reduced for both AB and AC word-pairs**

We investigated memory performance for targets and competitors as a function of word-pair type. At the end of each retrieval block, memory performance for the competitors was tested (i.e., after the AB/AC target memory test, the AC/AB competitor memory was tested). A two-way repeated-measures ANOVA, with the factors Word-Pair (AB vs. AC word-pairs) and Item Status (Target vs. Competitor) was performed. The analysis showed a significant effect of Word-Pair [*F*(1, 29) = 9.54, *p* = 0.004, $\eta_{p}^{2}$ = 0.25] – memory for AB was higher than memory for AC word-pairs. The effect of Item Status [*F*(1, 29) = 16.35, *p* < 0.001, $\eta_{p}^{2}$ = 0.36] was also significant – participant’s memory retrieval was better for targets compared with competitors, as would be expected as competitors were to be retrieved after prior retrieval of targets. There was no significant interaction between the two factors [*F*(1, 29) = 0.41, *p* = 0.53].

**Supplementary Note 4: Target context reinstatement for competitive retrieval versus non-competitive retrieval**

Differences in the temporal dynamics of context reinstatement during non-competitive and competitive retrieval were examined in a direct statistical comparison. We investigated 1) if target context reinstatement was stronger for non-competitive retrieval in the word-cue time window compared with competitive retrieval and 2) if target context reinstatement differed in the probe time window for competitive and non-competitive retrieval. A repeated-measure ANOVA including the factors Time-Window (Cue vs. Probe time windows) and Word-Pair (DE vs. AB vs. AC word-pairs) was performed on the classifier evidence for target context on successful trials. Classifier evidence was extracted separately for the word-cue and probe time windows for the time bins showing above-chance classification of target context and with a significantly stronger effect for successful compared with unsuccessful trials (using a leave-one-subject out approach). The ANOVA showed a significant interaction between Time-Window and Word-Pair [*F*(2, 54) = 6.75, *p* = 0.002, $\eta_{p}^{2}$ = 0.20]. Planned pairwise comparisons show that classifier evidence for target context reinstatement was indeed stronger in the word-cue time window during successful DE non-competitive retrieval compared with successful AC competitive retrieval [*t*(27) = 2.06, *p* = 0.049, *d* = 0.39]. Interestingly, the evidence for target context reinstatement for DE and AB word-pairs was comparable [*t*(29) = 0.84, *p* = 0.41, *d* = 0.15], which is reasonable given that there was no behavioral evidence of retroactive competition. In the probe time window, the evidence for target context reinstatement was stronger during successful AC and AB competitive retrieval [*t*(27) = 2.58, *p* = 0.016, *d* = 0.48; *t*(29) = 2.76, *p* = 0.010, *d* = 0.50] compared with successful DE non-competitive retrieval.

**Supplementary Note 5: DE, AB and AC movie reinstatement for non-competitive retrieval as a function of successful and unsuccessful retrieval**

To further explore context reinstatement modulations in DE non-competitive retrieval, we examined classifier evidence as a function of DE, AB, and AC movie and retrieval success (successful vs. unsuccessful). Classifier evidence was extracted for the three time windows showing context reinstatement significantly different from chance for successful DE target retrieval (we use a leave-one-subject out approach to select specific time points for each participant). **Figure S1** shows classification accuracy over the different conditions for these three time windows. A *t*-test contrasted classification accuracy against chance (33.3%) for each of the conditions. The only significant difference observed was the one already reported in the main analysis, that is, classification accuracy for DE movie is significantly higher than chance for successful DE retrieval in the early cue-time window [*t*(26) = 2.33, *p* = 0.028, *d* = 0.45]. All the other comparisons were not statistically significant (all *p*s > 0.12). Additionally, we ran a paired sample *t*-test contrasting successful and unsuccessful memory retrieval for each of the conditions. Again, the only significant difference observed was the one previously reported in the main analysis, that is, stronger DE context classification in the cue time window for successful compared with unsuccessful memory retrieval [*t*(26) = 2.42, *p* = 0.023, *d* = 0.46]. All the other comparisons were not statistically significant (all *p*s > 0.15).

**
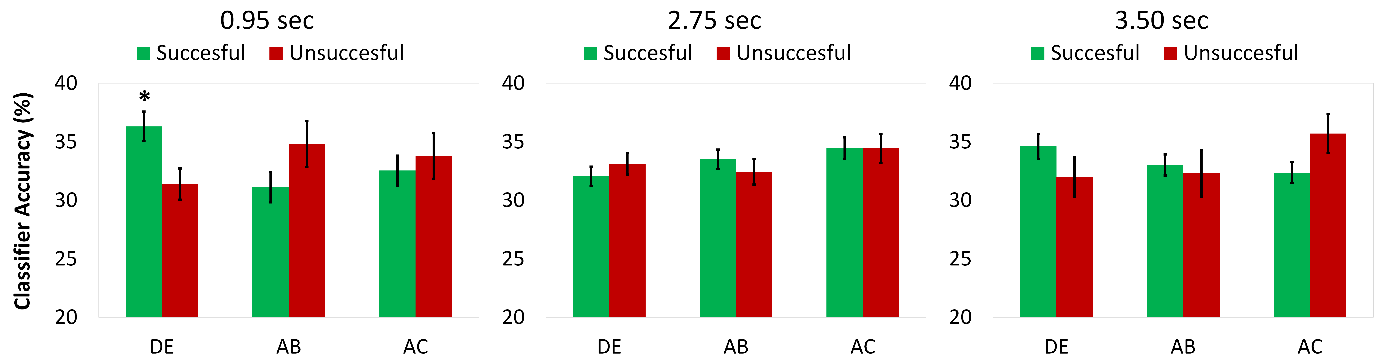
**

**Figure S1.** Averaged classification accuracy for DE, AB, and AC context reinstatement as a function of successful retrieval. The time-bins depicted correspond to the ones where reliable neural reinstatement was observed for successful trials. Highlighted classification evidence (*) indicates that classification accuracy was significantly different from chance. Error bars represent the standard error (SE) of the mean.

**Supplementary Note 6: Competitive retrieval is associated with an early increase in theta activity**

Previous studies indicate that memory interference is associated with increased frontal theta activity with an early onset (e.g., Bramão and Johansson 2017; Hanslmayr and others 2010; Staudigl and others 2010; Waldhauser and others 2012). To corroborate this prior finding, we contrasted AC and AB competitive retrieval with DE non-competitive retrieval with a cluster-based permutation analysis in the theta band (3-6 Hz) in the cue time window (between 0.1-0.6 after trial onset), and in the probe time window (between 2.1-2.6 after trial onset). Our data revealed significantly increased theta during successful AC competitive retrieval in the cue (*p* = 0.02, *d* = 0.35) and in the probe time window (p = 0.03, *d* = 0.55). For successful AB retrieval, the data only showed a significant increase in theta in the probe time window (*p* = 0.03, *d* = 0.54) (see **Figure S2**).

Next, we conducted an exploratory correlation analysis, examining the relationship between this effect and behavioral evidence for proactive interference (i.e., we subtracted, for each participant, the AC retrieval performance from the DE). We observed a significant correlation for the word-cue theta effect observed for successful AC retrieval (Pearson Correlation *r* = 0.43, *p* = 0.02, uncorrected), but not for the probe-period theta effect observed successful AC retrieval (Pearson Correlation *r* = 0.10, *p* = 0.61, uncorrected). Interpretative caution is warranted here given our small sample size (n = 30), which is known to increase false positive rates and give rise to over-estimated effect sizes.

These outcomes during the word-cue period corroborate the preceding findings by showing memory competition effects in the theta band, which may reflect either the simultaneous reactivation of the two memory traces associated with a given cue or between-trace competition for reactivation. The theta effects observed in the probe time window overlap with what is typically reported for successful episodic memory retrieval (Herweg and others 2020).

Notice, however, that the topography of the effects observed here did not show the typical pattern of being restricted to mid-anterior sites, as the distribution also involved posterior sites. Previous studies have mainly used word-list paradigms. In the current study, retrieval competition can be driven not only by the word associates, but also by the rich movie contexts, which may affect the topography of the effect.

**
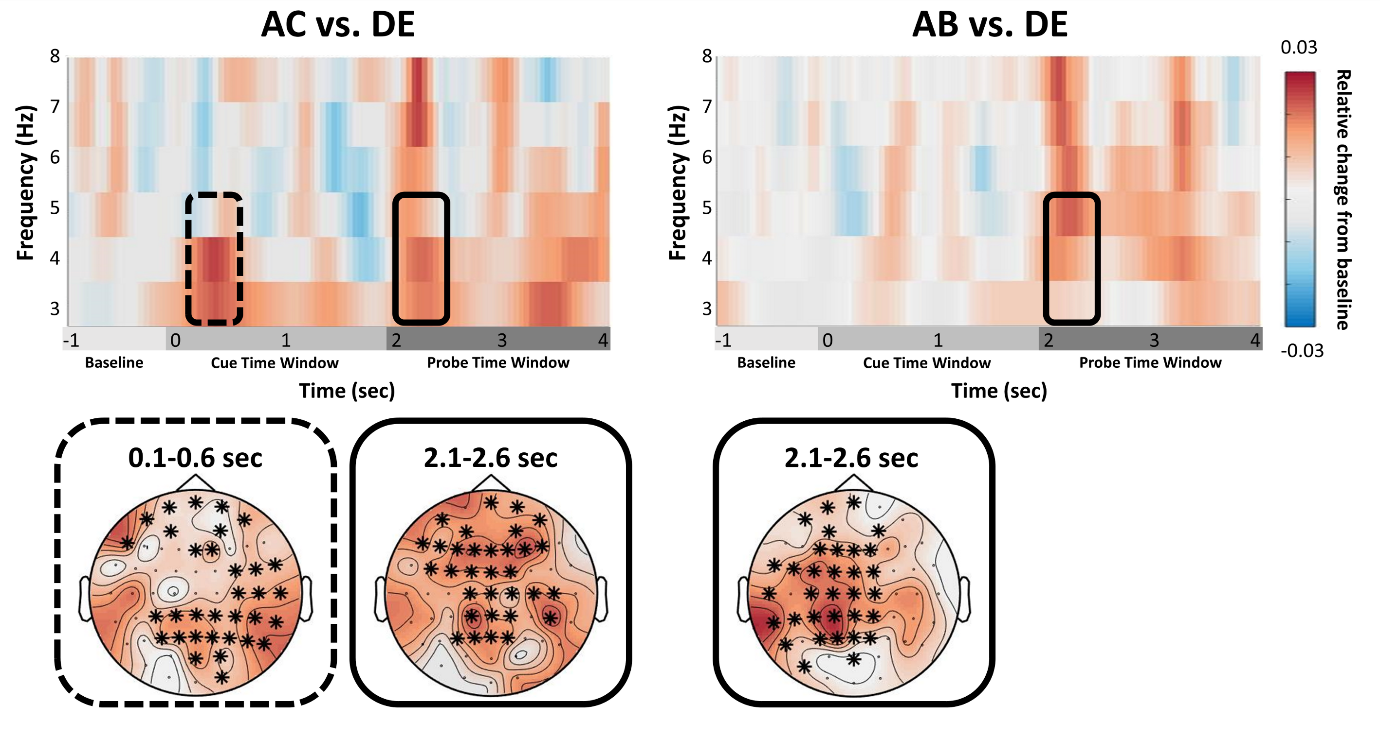
**

**Figure S2.** **Time-frequency representations of successful target retrieval.** Time-frequency representations for AC competitive retrieval vs. DE non-competitive retrieval and for AB competitive retrieval vs. DE non-competitive, for successful retrieval trials. On the upper row is shown the TFRs from representative channels. The areas where the effect was tested and for which a significant effect was observed are highlighted. On the lower row is shown the corresponding topography for the significant effects. Electrodes that reached significance are highlighted (*).

**Supplementary Note 7: Target context reinstatement in word-cue time window during AB retrieval**

To further understand the absence of target context reactivation during successful AB retrieval in the word-cue time window, we conducted an exploratory analysis, restricted to the time bins (1.1-1.3 s after word-cue onset) for which we observed classifier accuracy below chance during AC retrieval. In this analysis, we explored the evidence for target context retrieval during successful AB retrieval as a function of the encoding block type. Three types of blocks were used in the experiment: AB word-pairs could be presented in the beginning of the block, immediately followed (1) by the AC word-pairs (AB, AC and DE) or (2) by the DE word-pairs (AB, DE and AC); or (3) AB word-pairs could be presented after the DE word-pairs and followed by the AC word-pairs’ presentation (DE, AB and AC). If our task resulted in the encoding of AB association that are at least somewhat susceptible to interference from subsequent AC events, then target context reactivation for the AB word-pairs would be more likely to occur when the AB word-pairs are presented in the beginning of the block and are not immediately followed by the AC word-pairs (i.e., they are more temporally distinct, as in the AB, DE, AC block type). Corroborating this prediction, we observed significant above chance (33.3%) classification for AB target context reactivation in the AB, DE, AC block type [Mean ± SD = 38.8 ± 11.2, *t*(29) = 2.66, *p* = 0.013, *d* = 0.48], but not for the other two types of blocks [DE, AB, AC: Mean ± SD = 32.4 ± 12.0; AB, AC, DE: Mean ± SD = 33.3 ± 12.2; *p*s > 0.7].

**Supplementary Note 8: Context reinstatement as a function of memory for the AB/AC triplet**

We investigated if modulations in context reactivation, as retrieval unfolds, relates to the long-term accessibility of the two discrete events, i.e., the target and the other associate. To have enough trials, AB and AC retrieval conditions were collapsed and retrieval trials were separated into three categories: 1) remembering both the target and the associate; 2) retrieving the target memory but failing to retrieve the associate; and 3) failing to retrieve the current target but remembering the associate. The classifier, built with the encoding data, was applied throughout the retrieval epoch to detect the target context movie in these three types of trials in a subsample of participants with enough trials. **Figure S3** shows the outcomes of this analysis. During the word-cue time window, we observed classifier accuracy significantly below chance when the other associate memory trace was later remembered [Only associate remembered: Mean ± SD at 1.45 s = 28.37 ± 10.4, *t*(25) = -2.41, *p* = 0.023, *d* = -0.47, threshold t-value = -2.2; Both target and associate remembered: Mean ± SD at 1.3 s = 30.5 ± 6.4, *t*(28) = -2.37, *p* = 0.025, *d* = -0.44, threshold t-value = -2.16]. However, during the probe time window, the target’s encoding context was reinstated when the current target memory trace was remembered [Only target remembered: Mean ± SD at 0.55 s = 37.72 ± 11.2, *t*(28) = 2.12, *p* = 0.043, *d* = 0.39, threshold t-value = 2.1; Both target and associate remembered: Mean ± SD at 0.65 s = 36.65 ± 7.6, *t*(28) = 2.3, *p* = 0.028, *d* = 0.43, threshold t-value = 2.1].

Next, we investigated how target and associate context reactivation relates to the long-term fate of the associated memory trace. A two-way repeated-measures ANOVA, with the factors Memory Performance (Both target and associate vs. Only target vs. Only associate) and Context (target vs. associate) was computed for the cue and for the probe time window. A significant two-way interaction between Memory Performance and Context was observed in both analysis [Cue-Time Window: *F*(2,46) = 4.87, *p* = 0.012, $\eta_{p}^{2}$= 0.18; Probe Time Window: *F*(2,46) = 4.87, *p* = 0.017, $\eta_{p}^{2}$= 0.16]. Planned pairwise comparisons showed that, in the cue-time window, associate context reactivation was stronger compared with target reactivation when participants later remembered the associate [Both target and associate: *t*(28) = 3.12, *p* = 0.004, *d* = 0.58; Only associate: *t*(25) = 2.62, *p* = 0.015, *d* = 0.51; Only target: *t*(28) = 0.11, *p* = 0.92, *d* = 0.02]. Additionally, target context reactivation was stronger when participants only remembered the current target memory [Only target vs. Both target and associate: *t*(27) = 2.28, *p* = 0.03, *d* = 0.43; Only target vs. Only associate: *t*(24) = 3.03, *p* = 0.006, *d* = 0.61; Both target and associate vs. Only associate: *t*(24) = 1.23, *p* = 0.23, *d* = 0.25]. On the other hand, in the probe time window, target context reinstatement was stronger if participants remembered the current target memory [Both target and associate vs. Only associate: *t*(24) = 2.13, *p* = 0.044, *d* = 0.43; Only target vs. Only associate: *t*(24) = 2.72, *p* = 0.012, *d* = 0.55; Both target and associate vs. Only target: *t*(27) = 0.82, *p* = 0.42, *d* = 0.15].


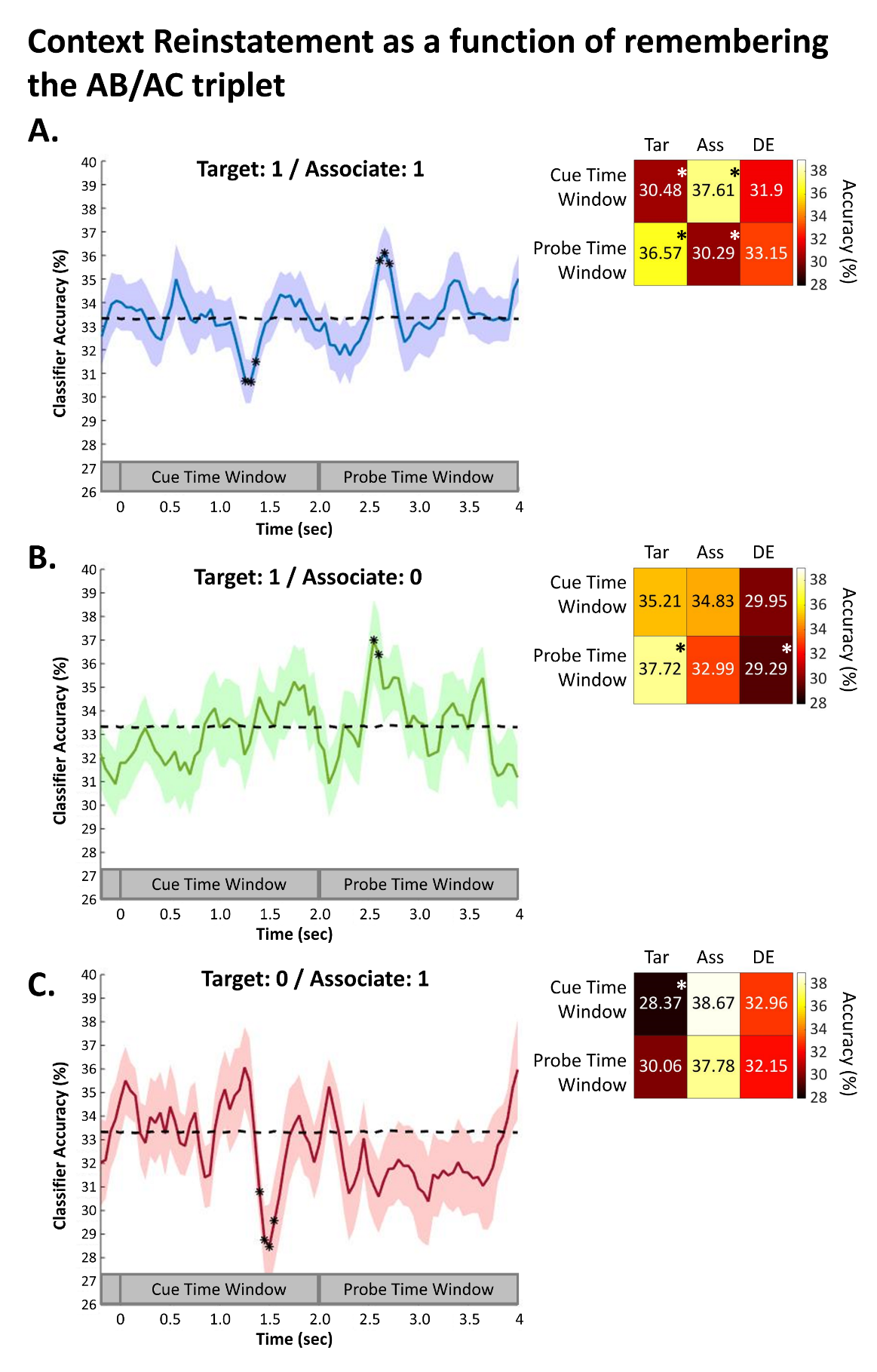


**Figure S3. Classification results as a function of remembering the target and the associate.** The classifier trained at encoding to discriminate between the three context movies was applied during the time course of competitive retrieval to track the reactivation of the neural patterns associated with the encoding contexts (target vs. associate vs. DE non-competitive retrieval). Averaged classification accuracy for (**A.**) when both target and associate were successfully remembered; (**B.**) only the current target memory trace was remembered; (**C.**) only the other associate was remembered. Chance level is shown in black and was calculated by averaging 1000 iterations with shuffled data. Classification accuracy was calculated in relation to target context movie. For illustration purposes, the classification was smoothed using a moving average with a size of 0.1 s. To identify the time bins for which classification performance was reliably different from chance, we ran 1000 classifications for each participant with shuffled data. A t-test comparing classification performance against chance (33.3%) was conducted for each of these iterations. The distribution of the t-tests obtained with the shuffled data formed the non-parametric empirical null distribution and the 97.5 and 2.5 percentiles of this distribution were used as the significance threshold for a two-tailed test, which corresponds to a threshold of a significance level of 0.05. Classification accuracy was considered significant if the t-value obtained when comparing classification against chance (33.3%) was higher than the threshold t-value obtained in the permutation test (see methods for details). The confusions matrices display the averaged classifier accuracy observed in the cue and probe time windows. Highlighted (*) are the times bins for which classification performance was significantly different from chance. Tar=target; Ass=associate; DE= non-competitor retrieval.

**References**

Bramão I, Johansson M. 2017. Benefits and costs of context reinstatement in episodic memory: An ERP study. Journal of Cognitive Neuroscience 29:52-64.

Hanslmayr S, Staudigl T, Aslan A, Bäuml K-H. 2010. Theta oscillations predict the detrimental effects of memory retrieval. Cognitive, Affective & Behavioral Neuroscience 10:329–338.

Herweg NA, Solomon EA, Kahana MJ. 2020. Theta oscillations in human memory. Trends in Cognitive Sciences 24:208-227.

Staudigl T, Hanslmayr S, Bäuml K-H. 2010. Theta oscillations reflect the dynamics of interference in episodic memory retrieval. The Journal of Neuroscience 30:11356-11362.

Waldhauser GT, Johansson M, Hanslmayr S. 2012. Alpha/Beta oscillations indicate inhibition of interfering visual memories. The Journal of Neuroscience 8:1953-1961.
